# Revisiting Minocycline in Intracerebral Hemorrhage: Mechanisms and Clinical Translation

**DOI:** 10.3389/fimmu.2022.844163

**Published:** 2022-03-17

**Authors:** Ruiyi Zhang, V. Wee Yong, Mengzhou Xue

**Affiliations:** ^1^ The Departments of Cerebrovascular Diseases, The Second Affiliated Hospital of Zhengzhou University, Zhengzhou, China; ^2^ Henan Medical Key Laboratory of Translational Cerebrovascular Diseases, The Second Affiliated Hospital of Zhengzhou University, Zhengzhou, China; ^3^ Hotchkiss Brain Institute, University of Calgary, Calgary, AB, Canada; ^4^ Department of Clinical Neurosciences, University of Calgary, Calgary, AB, Canada

**Keywords:** intracerebral hemorrhage, minocycline, secondary brain injury, neuroinflammation, mechanisms

## Abstract

Intracerebral hemorrhage (ICH) is an important subtype of stroke with an unsatisfactory prognosis of high mortality and disability. Although many pre-clinical studies and clinical trials have been performed in the past decades, effective therapy that meaningfully improve prognosis and outcomes of ICH patients is still lacking. An active area of research is towards alleviating secondary brain injury after ICH through neuroprotective pharmaceuticals and in which minocycline is a promising candidate. Here, we will first discuss new insights into the protective mechanisms of minocycline for ICH including reducing iron-related toxicity, maintenance of blood-brain barrier, and alleviating different types of cell death from preclinical data, then consider its shortcomings. Finally, we will review clinical trial perspectives for minocycline in ICH. We hope that this summary and discussion about updated information on minocycline as a viable treatment for ICH can facilitate further investigations.

## Introduction

Intracerebral hemorrhage (ICH) accounts for 12–20% of all types of strokes. It afflicts over 2 million individuals worldwide annually and is associated with an unacceptably high (50%) mortality and disability (1–3). Of the survivors, the vast majority (over 70%) are dependent on functioning aids a year after the event (4). ICH can be induced by several varied causes such as hypertension, cerebral amyloid angiopathy, trauma, vascular malformations, tumors, pre-mature birth, and drugs (5, 6). A study of global disease burden showed that the number of ICH cases has increased by 47% in the past 20 years, mostly afflicting low- and middle-income countries (7). Despite the obvious need to improve the prognosis of ICH, effective therapies have not emerged. However, there is a strong appreciation that ICH-induced neuroinflammation helps drive the progression of secondary brain injury in ICH. Hence, much research has been devoted to discovering efficacious agents to curb the neuroinflammation that promotes secondary brain injury. One such agent is minocycline.

Minocycline is a second-generation tetracycline derivative with a long history as an antibiotic since its approval by the FDA in a capsule form as minocin (8) in 1971. It stands apart from other tetracyclines by its high lipophilicity so that it has good penetration properties into the CNS (9). It protects against iron-mediated neurotoxicity in cell culture where other tetracyclines are ineffective (10). Minocycline has a long track record in stroke therapeutics, as the earliest reports over 20 years ago showed that this medication is neuroprotective in models of focal and global ischemic stroke (11–13). Since then, minocycline has demonstrated utility in many models of neurological diseases (14), and it has efficacy in clinical trials of patients with early multiple sclerosis (15) and traumatic spinal cord injury (16).

**Figure 1:**
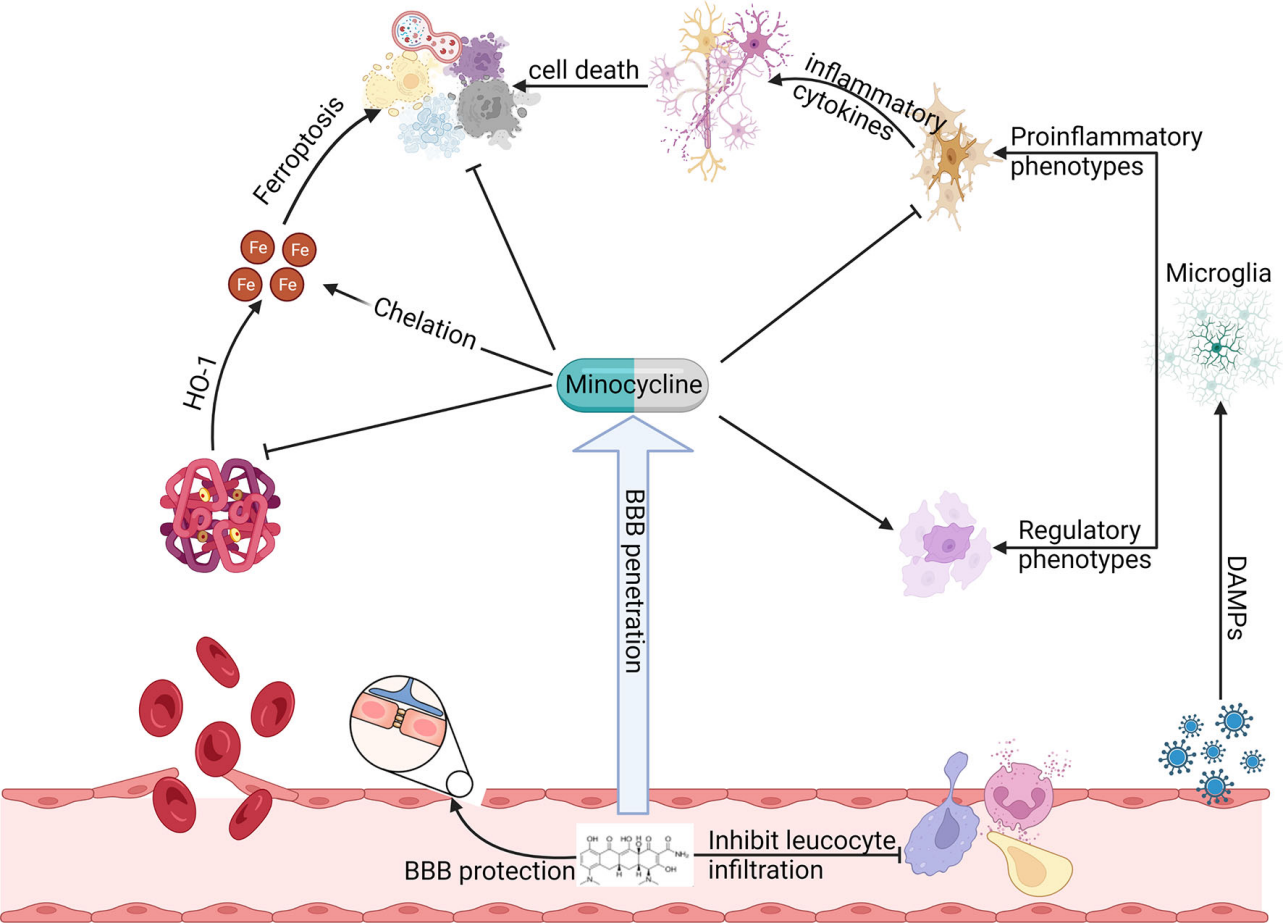
Preclinical studies have observed that minocycline can function at multiple steps of ICH induced secondary brain injury to produce neuroprotection. Minocycline inhibits HO-1 activity, chelates iron, alleviates oxidative stress, reduces various types of cell death, preserves BBB integrity, regulates leukocyte function, and inhibits proinflammatory microglia while promoting its regulatory phenotype.

Minocycline has broad-spectrum mechanisms that suggest its potential importance in ICH (**Figure 1**). This review intends to provide updated information on mechanisms of minocycline that afford potential utility in ICH. We will first discuss new insights into the protective mechanisms of minocycline for ICH including reducing iron-related toxicity, maintenance of integrity of the blood-brain barrier, and alleviating cell death. We will then consider its shortcomings. Finally, we will review clinical trial perspectives for minocycline in ICH.

## Mechanisms of Minocycline in Alleviating Injury Post-ICH

### Attenuation of Iron-Induced Neurotoxicity and Ferroptosis

After ICH, erythrocytes in the hematoma are lysed, releasing hemoglobin and heme into the injury site (17). These are then degraded into biliverdin, carbon monoxide, and iron. The accumulation of intracerebral iron after ICH begins within 24 h and peaks at 7 days (18), which can lead up to 3-fold increase of non-heme iron in the brain of rats; this elevation persists for at least a month after (19). Ample evidence suggests that iron overload is a prominent factor in the secondary injury of ICH; it promotes oxidative injury, brain atrophy, and long-term neurological deficit (18–23). High level of serum ferritin, an iron binding protein, is an independent risk factor related to severe brain edema and unsatisfactory prognosis (24, 25). Iron from hemoglobulin and heme can also be accumulated in microglia/macrophage by phagocytosis, resulting in their further activation and subsequent release of inflammatory mediators as well as free iron (26, 27). Notably, excessive ferrous iron can trigger Fenton reaction and the generation of the highly reactive radical, hydroxyl radical, and give rise to a type of cellular iron-dependent lethal lipid peroxidation of membrane polyunsaturated fatty acids, ferroptosis (28). Ferroptosis is a form of non-apoptotic regulated cell death distinguished from other types of cell death such as apoptosis, necrosis, pyroptosis, necroptosis, parthanatos, autophagy, Ca+ influx induced cell death (29, 30).

Neuronal and glial cells can be very sensitive to ferroptosis and glutamate-induced excitotoxicity after ICH (29, 31, 32), they literally work together in different steps of oxidative injury. The excess extracellular glutamate causes imbalanced gradient of concentration between different sides of cytomembrane in the ICH brain, which hampers the function of cystine/glutamate antiporter system $X_{\text{c}}^{-}$ , leading to scarcity of intracellular cysteine and decreased synthesis of glutathione, an important substrate of glutathione peroxidase to generate antioxidative reaction (33–36). Reduced cleaners (glutathione) need to cope with increasing burden (Fe^2+^ induced peroxide), which leads to devastating membrane peroxidation, or so called ferroptosis. Minocycline has been observed to alleviate glutamate-induced excitotoxicity, calcium influx and enhance cell survival in cultured neurons, but specific mechanism is not elucidated yet (37).

Things are clearer for iron, data show that applying iron chelators such as deferoxamine, or using specific inhibitors of ferroptosis such as ferrostatin-1 and N-acetylcysteine, improve neuron survival *in vitro* and vivo, reduce brain injury and improve rehabilitation of neurological functions in animal models of ICH (19, 30, 32, 38–40). But such compounds may have less prospect for clinical translation as they have unclear capacity to cross the blood-brain barrier and have scarce clinical safety data compared with minocycline. On the contrary, also as an iron chelator, minocycline can pass BBB easily and had well documented safety record (41).

Minocycline’s iron chelator properties (42) might be inferred from clinical work at the beginning, where the absorption of minocycline is significantly reduced with simultaneous administration of iron (43); also, skin hyperpigmentation occurs in patients taken long-term minocycline which is a precipitation of minocycline-iron complex (44). Chen-Roetling et al. firstly reported that minocycline has the capacity to attenuate iron-induced neurotoxicity in cortical cell cultures (45). They demonstrate that minocycline has better iron-chelation ability than deferoxamine under concentrations of 100 μM *in vitro* (45). Moreover, minocycline reduces lipid peroxidation as determined by low malondialdehyde (MDA) level and elevates ferritin level to assist in iron clearance while deferoxamine cannot (45).

Other research supports the mitigation of lipid peroxidation by minocycline, such as in rodent models of spinal cord injury and traumatic brain injury (46, 47). The results show that minocycline mitigates ferroptosis directly through antioxidative effects besides iron chelation. Zhao et al. tested minocycline on iron overload in the autologous blood model of ICH. Their results show that minocycline reduces serum total iron and brain non-heme iron as well as levels of ferritin, transferrin, transferrin receptor, and ceruloplasmin on day 3 and 7 after ICH (48). The ferritin data seems contradictory to the results *in vitro* mentioned above. But such divergence may be caused by different testing timepoints and environments of brain tissue and cell culture. More importantly, minocycline prevents cell death from ICH injury on day 3 and 7 post ICH injury, and promotes neuronal survival and BBB integrity at 24 hours after ferrous iron injection (48). They also report that minocycline reduces heme oxygenase-1 (HO-1) expression and alleviates brain edema 3 days after ferrous iron injection (49). HO-1 contributes to the increase of iron concentration after ICH for it degrades heme into biliverdin, carbon monoxide, and iron, which may exacerbate brain injury (50).

But other research proposes that HO-1 can be protective especially in the long term (51–53). The role of HO-1 in ICH still remains to be resolved, but inhibitors of HO-1 in the acute phase should be beneficial from the results we can gather (50, 53). Using magnetic resonance imaging (MRI), specifically T2-weighted, T2* gradient-echo combined with R2^*^ mapping in ICH rats, Cao et al. quantified iron deposition and found that minocycline reduced ICH induced iron overload as well as decreased lesion volume and improved neurological functions in 18 month-old aged rats at 7 and 28 days post injury (54–56). But such a technique has restrictions on determining iron content at certain phases of ICH (57). Recently, Yang et al. used quantitative susceptibility mapping (QSM) of MRI combined with diffusion tensor imaging (DTI) and concluded that minocycline reduced iron overload and white matter injury on day 28 in a minipig ICH model, correspondent with decreased brain edema, prevention of ventricle enlargement and improved functional prognosis (58).

**Figure 2:**
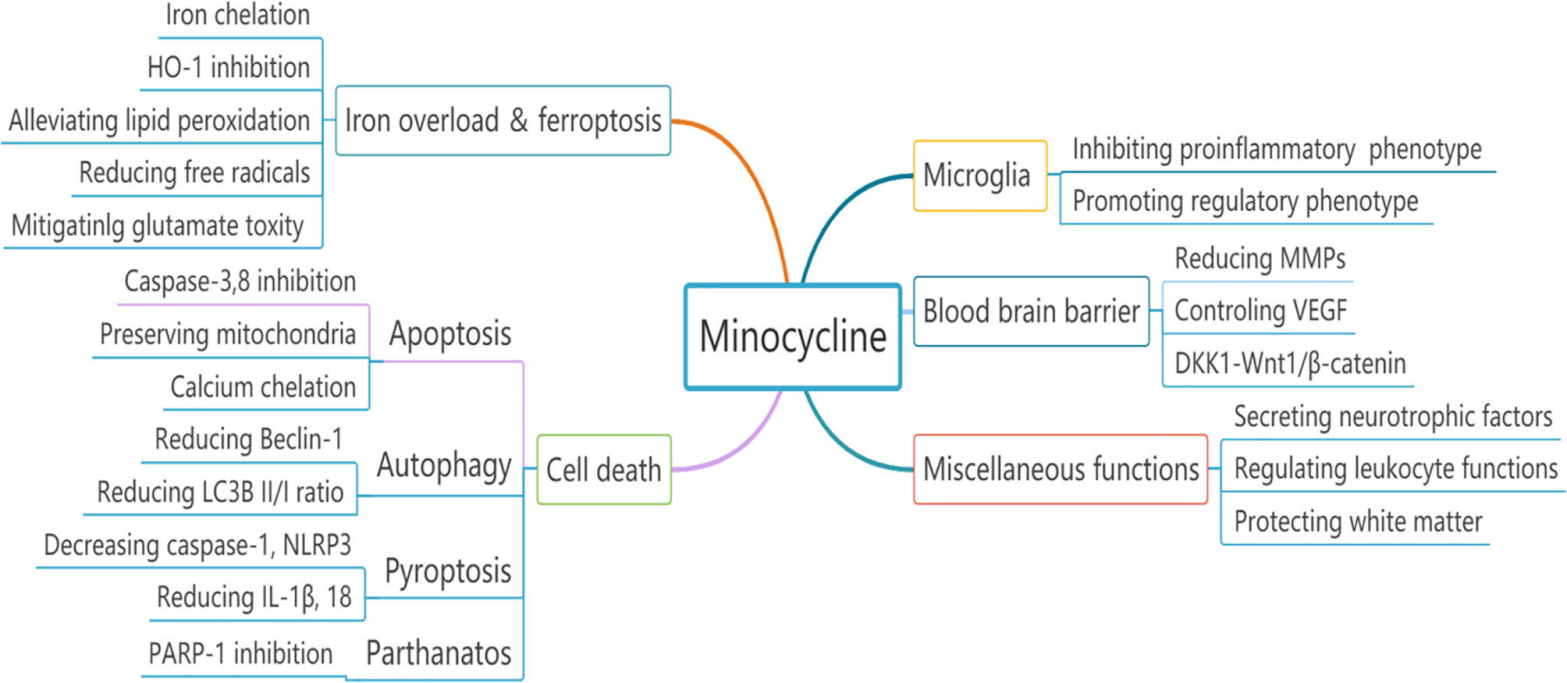
The neuroprotective effects of minocycline in experimental ICH could be attributed to multiple divergent mechanisms.

Overall, minocycline alleviates iron-related brain injury through iron chelation, ferroptosis antagonism and HO-1 inhibition in different ICH models including that caused by direct iron injection (**Figure 2**).

### Stabilization of Blood-Brain Barrier

The blood-brain barrier composed of capillary endothelial cells, capillary basement membrane, pericyte, and astrocyte end-feet is a highly selective semipermeable structure that maintains homeostasis and normal brain function (59, 60). This crucial structure is disrupted severely after ICH through multiple mechanisms of secondary injury. Following collagenase induced ICH of rats, BBB breakdown happens rapidly in 30 minutes and this status of hyperpermeability remains 5 hours to a lower but still disrupted level at 7 days (61). As for the autologous blood ICH model in rats, there is no BBB disruption detected in the first 4 hours while progressive damage shows up from 12 to 48 hours (62). In pigs, BBB disruption is also not observed in the early phase (1 to 8 hours after autologous injection) but evident by 24 hours (63–65). In ICH patients, data shows that some of them suffer continuous extravasation of contrast agents during the first 24 hours after onset (66, 67); there is also delayed exudation which may share the same condition of BBB disruption as animal models (68, 69). Such results indicate that BBB disruption could be a major cause of brain edema and hematoma expansion which can lead to the devastating consequence of midline shift and hernia. The disruption of BBB also leads to more leukocyte infiltration which exacerbates inflammation and brain injury undoubtedly.

Minocycline can attenuate ICH induced secondary BBB disruption through different pathways. Firstly, the matrix metalloproteinase (MMP) family including MMP-2, -3, -7, -9, and especially MMP-12 significantly increase after collagenase induced ICH which leads to the degradation of the extracellular matrix and capillary endothelial basal membrane of BBB (70–73). Xue et al. showed that increased gelatinolytic activity (likely of matrix metalloproteinase-2 and -9) was observed after 6 and 24 hours in the autologous blood-induced ICH mice associated with disruption of BBB (74). MMPs may also directly destroy the proteins of endothelial tight junction such as claudin-5 and occludin according to research in other neurological conditions (75, 76).

Several studies demonstrate that MMP-9 level is related to hematoma expansion, perihematomal edema, and neurological deterioration (77) and the association has been found that increased plasma MMP-3 and MMP-9 levels may be responsible for worse outcome and prognosis (78). Wasserman et al. reported that intraperitoneal injection of minocycline with a dosage of 45 mg/kg at 6 hours, 1 and 2 days after collagenase induced ICH in rats dramatically decreased the expression of MMP-12, TNF-α, and neutrophil infiltration as well as reduced BBB permeability on day 3 after modeling (79). Other scholars also suggest that minocycline has the ability to reduce MMP-2 and -9 (80, 81). An upstream activator of MMPs, named extracellular matrix metalloproteinase inducer (EMMPRIN), minocycline suppressed its function in the early phase of collagenase injection, which might be responsible for the downstream reduction in levels of MMPs and maintenance of blood brain barrier obtained with minocycline treatment (82). Such an inhibitory effect of minocycline may also contribute to prevent demyelination, decrease MMP-activated inflammation, and reduce cell death related molecules apart from the drug maintaining BBB integrity and reducing leukocyte infiltration (71).

Vascular endothelial growth factor (VEGF) increases sharply in many ICH models at the acute phase which can result in BBB destruction (83–85). Although VEGF may be beneficial in the recovery phase, the rapid upregulation of VEGF can be associated with brain injury and elevated MMPs levels in several hemorrhagic diseases (86–89). VEGF can even induce bleeding and hemoglobin extravasation in some animal models (90, 91). Lee et al. discovered that minocycline can mitigate VEGF transfection induced ICH through downregulation of MMP-9 expression in mice 48 hours after adenovirus injection (91); while Shi et al. demonstrated minocycline to reduce VEGF expression, preserve BBB integrity, and increase nerve growth factor (NGF) and heat shock protein (HSP) 70-positive cells from day 1 to day 14 in rat collagenase model (85). At almost the same time, Wu et al. reported that brain edema is reduced by minocycline at day 3 in autologous blood induced ICH model, and improved functional recovery was observed from day 1 to day 28 (92). Recently, Wang et al. reported that BBB disruption peaked at day 3 after collagenase induced ICH and such pathology was alleviated by minocycline administration, synchronized with decreased neurological deficits in behavioral tests (93). Moreover, their results also indicate that BBB protective character of minocycline was partly attributed to increasing occludin level and inhibiting TNF, IL-6, and MMP-9 production through DKK1-Wnt1/β-catenin signaling pathway (93).

To sum up, minocycline is an effective compound to reduce ICH mediated BBB disruption. The therapeutic outcome of minocycline is reduced brain edema, lower hematoma enlargement, and decreased brain atrophy. There is improved functional rehabilitation through multiple and integrated mechanisms.

### Attenuation of Apoptosis, Autophagy and Pyroptosis

Apoptosis contributes to various diseases in the nervous system and has been studied for many years. The classical process of apoptosis can be summarized into two inter-related pathways, extrinsic and intrinsic. The extrinsic apoptotic way begins with the combination of specific immune mediators such as tumor necrosis factor (TNF), FasL, tumor necrosis factor related apoptosis-induced ligand (TRAIL), and different death receptors on the cytomembrane; these lead to the formation of death-inducing signaling complex (DISC) that activates caspase-8 and finally caspase-3, the executioner of apoptosis (94–97). Such apoptosis-related immune mediators can be secreted in large amounts in the condition of ICH. In addition, oxidative stress damage of DNA, mitochondrial membrane and protein, mediated by free radicals generated during ICH, ignites intrinsic apoptotic pathways. The change of mitochondrial membrane leads to translocation of a series of apoptotic factors including cytochrome C, SMAC/DIABLO (Second Mitochondria-derived Activator of Caspases/Direct IAP-Binding protein with Low PI) and others from mitochondria to the cytoplasm which then activate caspase-9 and -3 successively (98, 99). Moreover, the caspase-8 produced in the extrinsic way and calcium overload in ICH can also initiate the intrinsic pathway by cleaving Bid into tBid and activate caspase-12, respectively (100–103). Apoptotic cells after autologous induced ICH are numerous as detected by TUNEL (104, 105). Felberg et al. also provided evidence for apoptosis by visualizing cytochrome c leakage within neurons after ICH in rats (106).

Minocycline can alleviate apoptotic cell death after ICH through both the extrinsic and intrinsic pathways. Minocycline inhibits caspase-3 directly (107, 108). Minocycline also maintains the proper permeability of mitochondrial membranes and limits the release of cytochrome C, apoptosis-inducing factor (AIF), and SMAC/DIABLO that contribute to apoptosis (107, 109, 110). Minocycline elevates Bcl-2 which is a protective anti-apoptotic molecule; the inhibition of Bcl-2 expression impairs such protection of minocycline (111).

Evidence also supports that as a calcium chelator, minocycline suppresses calcium-dependent apoptosis through the calpain-caspase -12 pathway (112, 113). Moreover, due to the ability to inhibit microglia/macrophage activation and reducing leukocyte infiltration by preserve BBB stability as mentioned above, minocycline can mitigate apoptosis by reducing TNF and other apoptosis-related cytokines from the source. Wu et al. found that minocycline inhibits apoptosis in the autologous blood ICH model in rats by observing decreased levels of caspase-3 and -8 as well as reduced TUNEL positive cell counts on day 1, 3 and 7 (114). As for the collagenase model, there were fewer Fluoro-Jade C and TUNEL positive cells on day 3 after minocycline treatment comparing to controls, indicating ameliorated apoptosis and neurodegeneration (115).

Autophagy is a process where a cell degrades its proteins or deficient organelles in the autophagic vacuole of its cytoplasm (116, 117). Such a process preserves cellular functions in normal physiological conditions. But excessive autophagy can also contribute to cell death. In the process of ICH, autophagy can be induced by several ways including oxidative stress, inflammation, and accumulation of free iron (118). He et al. first discovered the existence of autophagy after ICH by observing dramatically increased cathepsin D and microtubule-associated protein light chain 3 (LC3) II/I ratio, which are considered as biomarkers of autophagy (119–122). Moreover, they discovered autophagic vacuoles containing parts of membrane and cytoplasm, which provided visual evidence directly of autophagy in ICH (119, 123). It is plausible that minocycline has the capability of inhibiting ICH-induced autophagy by its ability to reduce free radicals and inflammation, and by iron chelation. Wu et al. demonstrated that minocycline reduces Beclin-1 and LC3B II/I ratio on 1 day post injury thereby mitigating autophagy in a rat model of autologous blood induced ICH. But in their results, Cathepsin D level does not differ between treatment and control (114). In some other pathological conditions, minocycline shows the potential of facilitating autophagy which could be beneficial (124–127). Thus, more study is essential for exploring the specific relationship and mechanism between minocycline and post-ICH autophagy.

Pyroptosis, another form of caspase-1 dependent programmed cell death proposed recently, is involved in the pathology of secondary brain injury after ICH. Pyroptosis ignites when pathogen-associated molecular pattern (PAMPs) or danger-associated molecular pattern (DAMP) bond with nucleotide-binding oligomerization domain-like receptors (NLRs) (128, 129). Such a combination happens in the condition of cellular damage or infection, which initiates the generation of NLR-based multiprotein complex, as known as the inflammasome. NLR pyrin domain containing 3 (NLRP3) is the most typical inflammasome studied in various neurodegenerative conditions (130–134). In such pathological environments, NLRP3 is observed initially to accumulate in microglia and may contribute to microglial activation, leading to expression of caspase-1 and downstream activation of interleukin (IL) -1β and 18 (135, 136). These cytokines can result in cellular pore formation, osmotic welling, loss of membrane integrity, which in turn give rise to the release of cell lysis and pro-inflammatory molecules into the extracellular matrix; this exacerbates inflammation and injury (137, 138). Wu et al. relate pyroptosis in post-ICH brain injury through reduced IL-1β, MMP-9 levels, and improved BBB integrity and neurological functions by inhibiting caspase-1 in ICH model (139). Feng et al. demonstrated that the collagenase-induced ICH model has significantly more caspase-1 production compared to the sham group as well as elevated NLRP3 levels (140). Ma et al. also published similar results and additionally that inhibition of NLRP3 expression reduced caspase-1 and IL-1β production (141).

The NLR activation and inflammasome formation in ICH are considered stimulated by oxidative free radicals and erythrocyte lysis products including hemoglobin and hemin (142–145). Moreover, the production of NLRP3 may also involve N-methyl-D-aspartic acid receptor 1 (NMDAR1) activation by hemin (146). Thus, pyroptosis contributes to post-ICH brain injury in animal models. In recent, researchers reported that minocycline attenuates pyroptosis in monosodium glutamate-induced depressive rats by detecting decreased caspase-1, NLRP3 inflammasome, IL-1β, and IL-18 levels in minocycline-administrated animals (147). More in-depth research is needed on the effects of minocycline in the process of pyroptosis after ICH.

### Inhibition of Microglial Activation

Microglia constitute 5–10% of the cellular population within the normal brain, and they are the first and main line of defense to pathological conditions of the central nervous system (148). In response to threat signals, microglia can change morphologically and functionally, and migrate towards the lesion field. Unless specifically differentiated by lineage markers, microglia cannot be distinguished with monocyte-derived macrophages infiltrated into the injured brain parenchyma (149). Hence, they are also referred to as microglia/macrophages (M/M). The activation of M/M plays a very crucial role in post-ICH secondary brain injury, which is also known as a double-edged sword. M/M activation contributes to the scavenging of hematoma and cellular debris in the subacute phase; homoeostatic/regulatory (M2-like) M/M activity also promotes neurogenesis, remyelination and angiogenesis in the chronic phase (150–152). On the contrary, the activation of pro-inflammatory (M1-like) M/M phenotype triggered by ICH-primary injury dominates in the early acute phase, which can lead to the release of a series of inflammatory cytokines, chemokines, MMPs, free radicals, and other molecules that exacerbate neuroinflammation and enhance secondary brain injury (153–155).

Minocycline is considered as a typically microglial activation inhibitor as first reported by Yrjanheikki and colleagues in a forebrain ischemia model in gerbils (11). The direct inhibition of microglial activation by minocycline was later shown in tissue culture (156, 157). Moreover, Kobayashi et al. reported that minocycline did not restrain regulatory microglia while it inhibited the pro-inflammatory phenotype (158). The microglial inhibition by minocycline ameliorates M/M activation related brain injury, and improves functional outcome through divergent pathways, which has been substantiated by many pre-clinical studies in different ICH animal models (48, 70, 93, 159, 160).

### Other Mechanisms of Minocycline Related to Post-ICH Neuroprotection

Poly (ADP-ribose) polymerase-1 (PARP-1) activation promotes DNA repair under normal cellular homeostasis. However, overwhelming activation of PARP-1 in the condition of oxidative stress leads to cell death and inflammation. PARP-1 expression is also the requirement of parthanatos, a newly defined type of cell death. Although there is scarce information on parthanatos in ICH, the expression of PARP-1 is associated with ICH (161, 162). Minocycline directly inhibits PARP-1 and confers beneficial effects apart from anti-oxidation in animal models of other diseases (163–167). The same mechanism may apply to ICH-induced secondary brain injury and more exploration is needed.

Besides inhibiting MMPs and maintain BBB integrity to reduce leukocyte infiltration, minocycline may affect leukocytes directly. Parenti et al. reported that minocycline inhibited respiratory burst and transendothelial migration of isolated human polymorphonuclear cells (168). Kloppenburg et al. reported that minocycline inhibited proliferation and reduced production of pro-inflammatory cytokines including IL-2, interferon (IFN) -γ and TNF-α from T cells of patients with rheumatoid arthritis (RA) (169).

Moreover, minocycline was observed to alleviate white matter injury and improve neurological deficits after autologous blood or iron intra-caudate injection on day 7 (170). Yang et al. confirmed the effect of minocycline on mitigating post-ICH white matter injury through elevated myelin basic protein (MBP) levels on day 14 as well as reduced IL-1β, induced nitric oxide synthase (iNOS) and TNF-α production on day 3 in blood induced piglet ICH; these researchers also implicated the minocycline related protection of white matter could be attributed to transforming growth factor‐β (TGF‐β)/mitogen‐activated protein kinase (MAPK) signaling pathway (160). Previous studies have also reported the MAPK inhibition by minocycline (156, 171).

Hsp70s act as cellular sentinel chaperones, protecting cells from multiple deleterious proteotoxic stresses; while NGF, one of the first growth factors isolated, involves the neuronal proliferation, maintenance and survival (172, 173). Minocycline has been found to increase the number of cells expressing NGF and HSP70 7 days after collagenase induced ICH, which might contribute to neuroprotection and tissue regeneration (174). More importantly, minocycline may potentiate neurogenesis after ICH since increased DCX (marker of neuronal precursor cells) and Tuj-1 (marker of neural stem cells and mature neurons) positive cells were observed in treatment group 24 hours after autologous blood injection (175).

As for immunomodulation, minocycline is well documented as a microglial activation inhibitor that reduces the generation of pro-inflammatory microglia. However, a recent study showed that minocycline can even promote the polarization of regulatory microglia on 3 days post injury as well as the generation of brain-derived neurotrophic factors (BDNF) and neuronal progenitor cells *via* TrkB/BDNF pathway in the rat ICH model of autologous blood injection (176).

## Limitations of Minocycline

The neuroprotective effects of minocycline are mainly attributed to its property of anti-inflammation and reducing inflammation-related brain injury as described above. Inflammation is involved in promoting various neurological pathology. However, recent studies indicate that neuroinflammation can also be beneficial especially in the phase of recovery through promoting remyelination, axonal generation, neurogenesis, and angiogenesis, which are all essential for neurofunctional rehabilitation in later phase of ICH (177). For instance, antagonizing toll-like receptor 4 (TLR-4), an inflammation associated pattern recognition receptor, results in reduced neurogenesis, angiogenesis, and functional recovery in a rat model of ICH by 14 days (178). In addition, the microglial phagocytosis is crucial for the clearance of hematoma, myeline and cellular debris in order to ignite repair process in later phase of ICH (175, 179). But the microglia inhibition including some phagocytotic phenotypes by minocycline may barricade recovery when treated too long (180, 181). Hence, long-term administration of minocycline after ICH may inhibit the benefits of inflammation.

In many ICH-related preclinical studies that demonstrated the effectiveness of minocycline treatment, the first administration is very early, within 2 hours or simultaneously with the injury (48, 54, 58, 92, 170). Minocycline showed no effects on reducing lesion volume, neural death, neurological deficits when the first dose was given 3 hours after onset in the collagenase-induced ICH model in rats, although minocycline was found to still reduce microglial activation, neutrophil infiltration, MMP-12, and TNF-α levels (182–184). The crucial time period of initiating minocycline would need more investigation as this is important for the practicability of clinical translation. On the other hand, animals have a much higher metabolic rate (half life of drugs tend to be shorter than in humans) and ICH models cannot replicate all features of human ICH, or the integrated therapies received by patients. More clinical trials are necessary to determine the real therapeutic promise of minocycline.

## Clinical Trials and the Future

The first clinical trial related to minocycline and ICH blended patients with acute ischemia stroke (AIS) and ICH. Only 11 actual ICH patients were included, with 100 mg intravenous administration of minocycline within 24 hours of stroke onset, which was continued 12 hourly for a total of 5 doses; the regimen seems safe but not efficacious from this pilot study of small sample (185).

Fouda et al. performed the well-known MACH (Minocycline in Acute Cerebral Hemorrhage) trial which included 16 consecutive eligible patients. Eight of the patients received 400mg of intravenous minocycline within 24 hours after onset, followed by 400 mg oral daily for 4 days. Pharmacokinetic data found that such a dose regimen produced a concentration suitable for neuroprotection demonstrated in a previous study in AIS rats (186). However, the MACH trial did not find any difference in 90-days modified Rankin Scale (mRS), MMP-9, IL-6, iron, ferritin, total iron-binding capacity, lesion volume, and perihematomal edema (187, 188).

Chang et al. also presented their results of a pilot study of 20 ICH patients in total. Ten randomly selected patients were treated with a relatively high dose (10 mg/kg) of intravenous minocycline within 12h from onset of symptoms and daily for the next 5 days. There were no differences in clinical and radiological outcomes, but serum MMP-9 levels seem to be reduced by minocycline administration (189).

All three above studies demonstrated the safety of minocycline in treating ICH, but no effectiveness was elucidated. Malhotra et al. conducted a meta-analysis of randomized clinical trials on minocycline treatment for acute stroke. In the subgroup analysis, treatment for AIS displayed much more positive results than ICH (190).

Altogether, whether minocycline should be pursued further in ICH is unresolved, as the sample size of all three trials is small and not enough to be representative. Moreover, patients may get better outcomes if they received minocycline earlier, within 3 hours of onset from pre-clinical data mentioned above, and with more frequent administration of a higher dose (191). The experience in clinical trial of spinal cord injury can be used for reference: a dosing of 800 mg intravenous minocycline was given within 12 h of injury, subsequent doses were gradually lowered down by 100 mg every 12 h until 400 mg and then the dosage is maintained for 7 days in total; CSF concentrations were kept between 2–3 μg/mL in this case, which are neuroprotective concentrations tested *in vitro (* 14, 16). The functional recovery was improved in patients received minocycline over 1 year of follow-up comparing to placebo, and the dose regimen was well tolerated (16). In addition, it is worth noting that all the patients included in previous clinical trials did not receive any form of hematoma evacuation surgery or had bleeding volume under 30 ml, which is not the indication of traditional hematoma removal surgery. Thus, the combination of surgical process with concurrent minocycline may show some benefits in coming clinical trials. Luo et al. provided pre-clinical proof for probability of such combined treatment in the rat model of autologous blood injection induced ICH. They began to remove the hematoma 4 hours after onset by aspiration surgery and then injected within 5 hours minocycline-loaded human hair keratose hydrogel into the center of lesion. The gel was a newly synthesized material that released minocycline slowly and also absorbed iron. The minocycline load was at the microgram level to reduce the possibility of adverse reactions and its combination with hematoma evacuation produced the best outcome in reducing brain damage and improving neurological functions across the groups tested (192).

## Conclusion

Although an old drug, minocycline continues to be promising for ICH. Studies in preclinical models affirm its capacity to reduce ICH neuropathology. This is attributed to its mechanisms that counter the injurious events of ICH as detailed above, and to its rapid initiation of treatment after ICH in models. We contend that a large scale clinical trial of minocycline in ICH, using high concentration and rapid initiation of treatment, and combined with hematoma extraction, is still promising for the unmet need of recovery from disastrous ICH.

## Search Strategy and Selection Criteria

References for this Review were identified by searches in English of PubMed (National Library of Science) 1970 and Feb 10, 2022, for the term “intracerebral hemorrhage” and a second term, which was ‘minocycline’, ‘inflammation’, ‘microglia’, ‘iron’, ‘ferroptosis’, ‘apoptosis’, ‘autophagy’, ‘cell death’, ‘blood brain barrier’, ‘oxidative stress’, ‘MMPs’, or ‘clinical trial’. The final reference list was generated on the basis of relevance to the topics covered in this Review.

## Author Contributions

All authors listed have made a substantial, direct, and intellectual contribution to the work and approved it for publication.

## Funding

The authors acknowledge operating grant support from National Key Research and Development Program of China (grant no: 2018YFC1312200), the National Natural Science Foundation of China (grants no: 82071331, 81870942, and 81520108011), and from the Canadian Institutes of Health Sciences (VY).

## Conflict of Interest

The authors declare that the research was conducted in the absence of any commercial or financial relationships that could be construed as a potential conflict of interest.

## Publisher’s Note

All claims expressed in this article are solely those of the authors and do not necessarily represent those of their affiliated organizations, or those of the publisher, the editors and the reviewers. Any product that may be evaluated in this article, or claim that may be made by its manufacturer, is not guaranteed or endorsed by the publisher.

## References

[1] Cordonnier C Demchuk A Ziai W Anderson CS. Intracerebral Haemorrhage: Current Approaches to Acute Management. Lancet (2018) 392(10154):1257–68. doi: 10.1016/S0140-6736(18)31878-6 30319113

[2] Zhang R Bai Q Liu Y Zhang Y Sheng Z Xue M. Intracerebral Hemorrhage in Translational Research. Brain Hemorrhages (2020) 1(1):13–8. doi: 10.1016/j.hest.2020.02.003

[3] Chen L Chen T Mao G Chen B Li M Zhang H. Clinical Neurorestorative Therapeutic Guideline for Brainstem Hemorrhage (2020 China Version). J Neurorestoratology (2021) 8(4):232–40. doi: 10.26599/JNR.2020.9040024

[4] Van Asch CJ Luitse MJ Rinkel GJ van der Tweel I Algra A Klijn CJ. Incidence, Case Fatality, and Functional Outcome of Intracerebral Haemorrhage Over Time, According to Age, Sex, and Ethnic Origin: A Systematic Review and Meta-Analysis. Lancet Neurol (2010) 9(2):167–76. doi: 10.1016/S1474-4422(09)70340-0 20056489

[5] An SJ Kim TJ Yoon B-W. Epidemiology, Risk Factors, and Clinical Features of Intracerebral Hemorrhage: An Update. J Stroke (2017) 19(1):3. doi: 10.5853/jos.2016.00864 28178408 PMC5307940

[6] Mohr JP Overbey JR Hartmann A Kummer RV Al-Shahi Salman R Kim H. Medical Management With Interventional Therapy Versus Medical Management Alone for Unruptured Brain Arteriovenous Malformations (ARUBA): Final Follow-Up of a Multicentre, non-Blinded, Randomised Controlled Trial. Lancet Neurol (2020) 19(7):573–81. doi: 10.1016/S1474-4422(20)30181-2 32562682

[7] Kang D-W. Intracerebral Hemorrhage: Large Disease Burden But Less Therapeutic Progress. J Stroke (2017) 19(1):1–2. doi: 10.5853/jos.2016.00024 28178404 PMC5307943

[8] Minocycline (Minocin). Med Lett Drugs Ther (1972) 14(4):9–10. 4206603

[9] Möller T Bard F Bhattacharya A Biber K Campbell B Dale E. Critical Data-Based Re-Evaluation of Minocycline as a Putative Specific Microglia Inhibitor. Glia (2016) 64(10):1788–94. doi: 10.1002/glia.23007 27246804

[10] Faissner S Mahjoub Y Mishra M Haupeltshofer S Hahn JN Gold R. Unexpected Additive Effects of Minocycline and Hydroxychloroquine in Models of Multiple Sclerosis: Prospective Combination Treatment for Progressive Disease? Multiple Sclerosis J (2018) 24(12):1543–56. doi: 10.1177/1352458517728811 28857721

[11] Yrjänheikki J Keinänen R Pellikka M Hökfelt T Koistinaho J. Tetracyclines Inhibit Microglial Activation and are Neuroprotective in Global Brain Ischemia. Proc Natl Acad Sci (1998) 95(26):15769–74. doi: 10.1073/pnas.95.26.15769 PMC28119 9861045

[12] Yrjänheikki J Tikka T Keinänen R Goldsteins G Chan PH Koistinaho J. A Tetracycline Derivative, Minocycline, Reduces Inflammation and Protects Against Focal Cerebral Ischemia With a Wide Therapeutic Window. Proc Natl Acad Sci (1999) 96(23):13496–500. doi: 10.1073/pnas.96.23.13496 PMC23976 10557349

[13] Sheng Z Liu Y Li H Zheng W Xia B Zhang X. Efficacy of Minocycline in Acute Ischemic Stroke: A Systematic Review and Meta-Analysis of Rodent and Clinical Studies. Front Neurol (2018) 9:1103. doi: 10.3389/fneur.2018.01103 30619060 PMC6306456

[14] Yong VW Wells J Giuliani F Casha S Power C Metz LM. The Promise of Minocycline in Neurology. Lancet Neurol (2004) 3(12):744–51. doi: 10.1016/S1474-4422(04)00937-8 15556807

[15] Metz LM Li DK Traboulsee AL Duquette P Eliasziw M Cerchiaro G. Trial of Minocycline in a Clinically Isolated Syndrome of Multiple Sclerosis. New Engl J Med (2017) 376(22):2122–33. doi: 10.1056/NEJMoa1608889 28564557

[16] Casha S Zygun D McGowan MD Bains I Yong VW John Hurlbert R. Results of a Phase II Placebo-Controlled Randomized Trial of Minocycline in Acute Spinal Cord Injury. Brain (2012) 135(4):1224–36. doi: 10.1093/brain/aws072 22505632

[17] Bai Q Sheng Z Liu Y Zhang R Yong VW Xue M. Intracerebral Haemorrhage: From Clinical Settings to Animal Models. Stroke Vasc Neurol (2020) 5(4):388–95. doi: 10.1136/svn-2020-000334 PMC7804065 33376200

[18] Wu J Hua Y Keep RF Nakamura T Hoff JT Xi G. Iron and Iron-Handling Proteins in the Brain After Intracerebral Hemorrhage. Stroke (2003) 34(12):2964–9. doi: 10.1161/01.STR.0000103140.52838.45 14615611

[19] Hua Y Keep RF Hoff JT Xi G. Brain Injury After Intracerebral Hemorrhage: The Role of Thrombin and Iron. Stroke (2007) 38(2):759–62. doi: 10.1161/01.STR.0000247868.97078.10 17261733

[20] Huang F Xi G Keep R Hua Y Nemoianu A Hoff J. Brain Edema After Experimental Intracerebral Hemorrhage: Role of Hemoglobin Degradation Products. J Neurosurg (2002) 96(2):287–93. doi: 10.3171/jns.2002.96.2.0287 11838803

[21] Hua Y Nakamura T Keep RF Wu J Schallert T Hoff JT. Long-Term Effects of Experimental Intracerebral Hemorrhage: The Role of Iron. J Neurosurg (2006) 104(2):305–12. doi: 10.3171/jns.2006.104.2.305 16509506

[22] Nakamura T Keep RF Hua Y Nagao S Hoff J Xi G. Iron-Induced Oxidative Brain Injury After Experimental Intracerebral Hemorrhage. Acta Neurochir Suppl (2006) 96:194–8. doi: 10.1007/3-211-30714-1_42 16671453

[23] Wu G Xi G Huang F. Spontaneous Intracerebral Hemorrhage in Humans: Hematoma Enlargement, Clot Lysis, and Brain Edema. Acta Neurochir Suppl (2006) 96:78–80. doi: 10.1007/3-211-30714-1_19 16671430

[24] Mehdiratta M Kumar S Hackney D Schlaug G Selim M. Association Between Serum Ferritin Level and Perihematoma Edema Volume in Patients With Spontaneous Intracerebral Hemorrhage. Stroke (2008) 39(4):1165–70. doi: 10.1161/STROKEAHA.107.501213 18292378

[25] Peírez de la Ossa N Sobrino T Silva Y Blanco M Millán M Gomis M. Iron-Related Brain Damage in Patients With Intracerebral Hemorrhage. Stroke (2010) 41(4):810–3. doi: 10.1161/STROKEAHA.109.570168 20185788

[26] Kroner A Greenhalgh AD Zarruk JG dos Santos RP Gaestel M David S. TNF and Increased Intracellular Iron Alter Macrophage Polarization to a Detrimental M1 Phenotype in the Injured Spinal Cord. Neuron (2014) 83(5):1098–116. doi: 10.1016/j.neuron.2014.07.027 25132469

[27] Hu X Leak RK Shi Y Suenaga J Gao Y Zheng P. Microglial and Macrophage Polarization—New Prospects for Brain Repair. Nat Rev Neurol (2015) 11(1):56. doi: 10.1038/nrneurol.2014.207 25385337 PMC4395497

[28] Stockwell BR Angeli JPF Bayir H Bush AI Conrad M Dixon SJ. Ferroptosis: A Regulated Cell Death Nexus Linking Metabolism, Redox Biology, and Disease. Cell (2017) 171(2):273–85. doi: 10.1016/j.cell.2017.09.021 PMC5685180 28985560

[29] Dixon SJ Lemberg KM Lamprecht MR Skouta R Zaitsev EM Gleason CE. Ferroptosis: An Iron-Dependent Form of Nonapoptotic Cell Death. Cell (2012) 149(5):1060–72. doi: 10.1016/j.cell.2012.03.042 PMC3367386 22632970

[30] Zille M Karuppagounder SS Chen Y Gough PJ Bertin J Finger J. Neuronal Death After Hemorrhagic Stroke *In Vitro* and *In Vivo* Shares Features of Ferroptosis and Necroptosis. Stroke (2017) 48(4):1033–43. doi: 10.1161/STROKEAHA.116.015609 PMC5613764 28250197

[31] Yang WS Stockwell BR. Ferroptosis: Death by Lipid Peroxidation. Trends Cell Biol (2016) 26(3):165–76. doi: 10.1016/j.tcb.2015.10.014 PMC4764384 26653790

[32] Li Q Han X Lan X Gao Y Wan J Durham F. Inhibition of Neuronal Ferroptosis Protects Hemorrhagic Brain. JCI Insight (2017) 2(7):e90777. doi: 10.1172/jci.insight.90777 28405617 PMC5374066

[33] Yang WS SriRamaratnam R Welsch ME Shimada K Skouta R Viswanathan VS. Regulation of Ferroptotic Cancer Cell Death by GPX4. Cell (2014) 156(1-2):317–31. doi: 10.1016/j.cell.2013.12.010 PMC4076414 24439385

[34] Conrad M Friedmann Angeli JP. Glutathione Peroxidase 4 (Gpx4) and Ferroptosis: What's So Special About it? Mol Cell Oncol (2015) 2(3):e995047. doi: 10.4161/23723556.2014.995047 27308484 PMC4905320

[35] Gao M Monian P Quadri N Ramasamy R Jiang X. Glutaminolysis and Transferrin Regulate Ferroptosis. Mol Cell (2015) 59(2):298–308. doi: 10.1016/j.molcel.2015.06.011 26166707 PMC4506736

[36] Angeli JPF Shah R Pratt DA Conrad M. Ferroptosis Inhibition: Mechanisms and Opportunities. Trends Pharmacol Sci (2017) 38(5):489–98. doi: 10.1016/j.tips.2017.02.005 28363764

[37] Lu Y Yang Y Chen W Du N Du Y Gu H. Minocycline, But Not Doxycycline Attenuates NMDA-Induced [Ca2+]i and Excitotoxicity. Neuroreport (2021) 32(1):38–43. doi: 10.1097/WNR.0000000000001558 33252477

[38] Nakamura T Keep RF Hua Y Schallert T Hoff JT Xi G. Deferoxamine-Induced Attenuation of Brain Edema and Neurological Deficits in a Rat Model of Intracerebral Hemorrhage. J Neurosurg (2004) 100(4):672–8. doi: 10.3171/jns.2004.100.4.0672 15070122

[39] Xi G Keep RF Hoff JT. Mechanisms of Brain Injury After Intracerebral Haemorrhage. Lancet Neurol (2006) 5(1):53–63. doi: 10.1016/S1474-4422(05)70283-0 16361023

[40] Xiong X-Y Wang J Qian Z-M Yang Q-W. Iron and Intracerebral Hemorrhage: From Mechanism to Translation. Trans Stroke Res (2014) 5(4):429–41. doi: 10.1007/s12975-013-0317-7 24362931

[41] Frenzel T Lee CZ Kim H Quinnine NJ Hashimoto T Lawton MT. Feasibility of Minocycline and Doxycycline Use as Potential Vasculostatic Therapy for Brain Vascular Malformations: Pilot Study of Adverse Events and Tolerance. Cerebrovasc Dis (2008) 25(1-2):157–63. doi: 10.1159/000113733 PMC2605012 18212521

[42] Grenier D Huot M-P Mayrand D. Iron-Chelating Activity of Tetracyclines and its Impact on the Susceptibility of Actinobacillus Actinomycetemcomitansto These Antibiotics. Antimicrob Agents Chemother (2000) 44(3):763–6. doi: 10.1128/AAC.44.3.763-766.2000 PMC89761 10681353

[43] Leyden JJ. Absorption of Minocycline Hydrochloride and Tetracycline Hydrochloride: Effect of Food, Milk, and Iron. J Am Acad Dermatol (1985) 12(2):308–12. doi: 10.1016/S0190-9622(85)80041-4 3838321

[44] Geria AN Tajirian AL Kihiczak G Schwartz RA. Minocycline-Induced Skin Pigmentation: An Update. Acta Dermatovenerologica Croatica (2009) 17(2):123–6. 19595269

[45] Chen-Roetling J Chen L Regan RF. Minocycline Attenuates Iron Neurotoxicity in Cortical Cell Cultures. Biochem Biophys Res Commun (2009) 386(2):322–6. doi: 10.1016/j.bbrc.2009.06.026 PMC2782944 19523448

[46] Sonmez E Kabatas S Ozen O Karabay G Turkoglu S Ogus E. Minocycline Treatment Inhibits Lipid Peroxidation, Preserves Spinal Cord Ultrastructure, and Improves Functional Outcome After Traumatic Spinal Cord Injury in the Rat. Spine (2013) 38(15):1253–9. doi: 10.1097/BRS.0b013e3182895587 23370685

[47] Homsi S Federico F Croci N Palmier B Plotkine M Marchand-Leroux C. Minocycline Effects on Cerebral Edema: Relations With Inflammatory and Oxidative Stress Markers Following Traumatic Brain Injury in Mice. Brain Res (2009) 1291:122–32. doi: 10.1016/j.brainres.2009.07.031 19631631

[48] Zhao F Hua Y He Y Keep RF Xi G. Minocycline-Induced Attenuation of Iron Overload and Brain Injury After Experimental Intracerebral Hemorrhage. Stroke (2011) 42(12):3587–93. doi: 10.1161/STROKEAHA.111.623926 PMC3226873 21998050

[49] Zhao F Xi G Liu W Keep RF Hua Y. Minocycline Attenuates Iron-Induced Brain Injury. Acta Neurochir Suppl (2016) 121:361–5. doi: 10.1007/978-3-319-18497-5_62 26463975

[50] Wang J Doré S. Heme Oxygenase-1 Exacerbates Early Brain Injury After Intracerebral Haemorrhage. Brain (2007) 130(6):1643–52. doi: 10.1093/brain/awm095 PMC2291147 17525142

[51] Chen-Roetling J F Regan R. Targeting the Nrf2-Heme Oxygenase-1 Axis After Intracerebral Hemorrhage. Curr Pharm Des (2017) 23(15):2226–37. doi: 10.2174/1381612822666161027150616 PMC5474205 27799046

[52] Yin X-P Wu D Zhou J Chen Z-Y Bao B Xie L. Heme Oxygenase 1 Plays Role of Neuron-Protection by Regulating Nrf2-ARE Signaling Post Intracerebral Hemorrhage. Int J Clin Exp Pathol (2015) 8(9):10156–63. PMC4637538 26617723

[53] Zhang Z Song Y Zhang Z Li D Zhu H Liang R. Distinct Role of Heme Oxygenase-1 in Early-and Late-Stage Intracerebral Hemorrhage in 12-Month-Old Mice. J Cereb Blood Flow Metab (2017) 37(1):25–38. doi: 10.1177/0271678X16655814 27317654 PMC5363754

[54] Cao S Hua Y Keep RF Chaudhary N Xi G. Minocycline Effects on Intracerebral Hemorrhage-Induced Iron Overload in Aged Rats: Brain Iron Quantification With Magnetic Resonance Imaging. Stroke (2018) 49(4):995–1002. doi: 10.1161/STROKEAHA.117.019860 29511126 PMC5871578

[55] Wu G Xi G Hua Y Sagher O. T2* Magnetic Resonance Imaging Sequences Reflect Brain Tissue Iron Deposition Following Intracerebral Hemorrhage. Trans Stroke Res (2010) 1(1):31–4. doi: 10.1007/s12975-009-0008-6 PMC2930789 20811505

[56] Belayev L Obenaus A Zhao W Saul I Busto R Wu C. Experimental Intracerebral Hematoma in the Rat: Characterization by Sequential Magnetic Resonance Imaging, Behavior, and Histopathology. Effect of Albumin Therapy. Brain Res (2007) 1157:146–55. doi: 10.1016/j.brainres.2007.04.077 17543290

[57] Chang S Zhang J Liu T Tsiouris AJ Shou J Nguyen T. Quantitative Susceptibility Mapping of Intracerebral Hemorrhages at Various Stages. J Magn Reson Imaging (2016) 44(2):420–5. doi: 10.1002/jmri.25143 PMC4930428 26718014

[58] Yang Y Zhang K Yin X Lei X Chen X Wang J. Quantitative Iron Neuroimaging Can Be Used to Assess the Effects of Minocycline in an Intracerebral Hemorrhage Minipig Model. Trans Stroke Res (2020) 11(3):503–16. doi: 10.1007/s12975-019-00739-2 31696415

[59] Daneman R Prat A. The Blood-Brain Barrier. Cold Spring Harb Perspect Biol (2015) 7(1):a020412. doi: 10.1101/cshperspect 25561720 PMC4292164

[60] Ballabh P Braun A Nedergaard M. The Blood–Brain Barrier: An Overview: Structure, Regulation, and Clinical Implications. Neurobiol Dis (2004) 16(1):1–13. doi: 10.1016/j.nbd.2003.12.016 15207256

[61] Rosenberg GA Estrada E Kelley RO Kornfeld M. Bacterial Collagenase Disrupts Extracellular Matrix and Opens Blood-Brain Barrier in Rat. Neurosci Lett (1993) 160(1):117–9. doi: 10.1016/0304-3940(93)90927-D 8247322

[62] Yang G-Y Betz AL Chenevert TL Brunberg JA Hoff JT. Experimental Intracerebral Hemorrhage: Relationship Between Brain Edema, Blood Flow, and Blood-Brain Barrier Permeability in Rats. J Neurosurg (1994) 81(1):93–102. doi: 10.3171/jns.1994.81.1.0093 8207532

[63] Wagner KR Xi G Hua Y Kleinholz M de Courten-Myers GM Myers RE. Lobar Intracerebral Hemorrhage Model in Pigs: Rapid Edema Development in Perihematomal White Matter. Stroke (1996) 27(3):490–7. doi: 10.1161/01.STR.27.3.490 8610319

[64] Wagner KR Xi G Hua Y Zuccarello M de Courten-Myers GM Broderick JP. Ultra-Early Clot Aspiration After Lysis With Tissue Plasminogen Activator in a Porcine Model of Intracerebral Hemorrhage: Edema Reduction and Blood-Brain Barrier Protection. J Neurosurg (1999) 90(3):491–8. doi: 10.3171/jns.1999.90.3.0491 10067918

[65] Marchi N Rasmussen P Kapural M Fazio V Kight K Mayberg MR. Peripheral Markers of Brain Damage and Blood-Brain Barrier Dysfunction. Restor Neurol Neurosci (2003) 21(3–4):109–21. PMC4066375 14530574

[66] Brouwers HB Greenberg SM. Hematoma Expansion Following Acute Intracerebral Hemorrhage. Cerebrovascular Dis (2013) 35(3):195–201. doi: 10.1159/000346599 PMC3743539 23466430

[67] Hallevi H Abraham AT Barreto AD Grotta JC Savitz SI. The Spot Sign in Intracerebral Hemorrhage: The Importance of Looking for Contrast Extravasation. Cerebrovascular Dis (2010) 29(3):217–20. doi: 10.1159/000267842 20029193

[68] Delgado P Alvarez Sabin J Santamarina E Molina CA Quintana M Rosell A. Plasma S100B Level After Acute Spontaneous Intracerebral Hemorrhage. Stroke (2006) 37(11):2837–9. doi: 10.1161/01.STR.0000245085.58807.ad 17008613

[69] Murai Y Ikeda Y Teramoto A Tsuji Y. Magnetic Resonance Imaging—Documented Extravasation as an Indicator of Acute Hypertensive Intracerebral Hemorrhage. J Neurosurg (1998) 88(4):650–5. doi: 10.3171/jns.1998.88.4.0650 9525710

[70] Power C Henry S Del Bigio MR Larsen PH Corbett D Imai Y. Intracerebral Hemorrhage Induces Macrophage Activation and Matrix Metalloproteinases. Ann Neurol (2003) 53(6):731–42. doi: 10.1002/ana.10553 12783419

[71] Yong VW Power C Forsyth P Edwards DR. Metalloproteinases in Biology and Pathology of the Nervous System. Nat Rev Neurosci (2001) 2(7):502–11. doi: 10.1038/35081571 PMC7097548 11433375

[72] Aronowski J Zhao X. Molecular Pathophysiology of Cerebral Hemorrhage: Secondary Brain Injury. Stroke (2011) 42(6):1781–6. doi: 10.1161/STROKEAHA.110.596718 PMC3123894 21527759

[73] Wang J Doré S. Inflammation After Intracerebral Hemorrhage. J Cereb Blood Flow Metab (2007) 27(5):894–908. doi: 10.1038/sj.jcbfm.9600403 17033693

[74] Xue M Hollenberg MD Yong VW. Combination of Thrombin and Matrix Metalloproteinase-9 Exacerbates Neurotoxicity in Cell Culture and Intracerebral Hemorrhage in Mice. J Neurosci (2006) 26(40):10281–91. doi: 10.1523/JNEUROSCI.2806-06.2006 PMC6674619 17021183

[75] Lischper M Beuck S Thanabalasundaram G Pieper C Galla H-J. Metalloproteinase Mediated Occludin Cleavage in the Cerebral Microcapillary Endothelium Under Pathological Conditions. Brain Res (2010) 1326:114–27. doi: 10.1016/j.brainres.2010.02.054 20197061

[76] Yang Y Rosenberg GA. MMP-Mediated Disruption of Claudin-5 in the Blood–Brain Barrier of Rat Brain After Cerebral Ischemia. Methods Mol Biol (2011) 762:333–45. doi: 10.1007/978-1-61779-185-7_24 PMC4950933 21717368

[77] Florczak-Rzepka M Grond-Ginsbach C Montaner J Steiner T. Matrix Metalloproteinases in Human Spontaneous Intracerebral Hemorrhage: An Update. Cerebrovascular Dis (2012) 34(4):249–62. doi: 10.1159/000341686 23052179

[78] Li N Liu YF Ma L Worthmann H Wang YL Wang YJ. Association of Molecular Markers With Perihematomal Edema and Clinical Outcome in Intracerebral Hemorrhage. Stroke (2013) 44(3):658–63. doi: 10.1161/STROKEAHA.112.673590 23391772

[79] Wasserman JK Schlichter LC. Minocycline Protects the Blood–Brain Barrier and Reduces Edema Following Intracerebral Hemorrhage in the Rat. Exp Neurol (2007) 207(2):227–37. doi: 10.1016/j.expneurol.2007.06.025 17698063

[80] Hwang BY Appelboom G Ayer A Kellner CP Kotchetkov IS Gigante PR. Advances in Neuroprotective Strategies: Potential Therapies for Intracerebral Hemorrhage. Cerebrovascular Dis (2011) 31(3):211–22. doi: 10.1159/000321870 PMC3721946 21178344

[81] Katsuki H. Exploring Neuroprotective Drug Therapies for Intracerebral Hemorrhage. J Pharmacol Sci (2010) 114(4):366–78. doi: 10.1254/jphs.10R05CR 21081835

[82] Liu Y Li Z Khan S Zhang R Wei R Zhang Y. Neuroprotection of Minocycline by Inhibition of Extracellular Matrix Metalloproteinase Inducer Expression Following Intracerebral Hemorrhage in Mice. Neurosci Lett (2021) 764:136297. doi: 10.1016/j.neulet.2021.136297 34666120

[83] You P Lin M Li K Ye X Zheng J. Normobaric Oxygen Therapy Inhibits HIF-1α and VEGF Expression in Perihematoma and Reduces Neurological Function Defects. Neuroreport (2016) 27(5):329–36. doi: 10.1097/WNR.0000000000000542 26872098

[84] Hu S Wu G Zheng J Liu X Zhang Y. Astrocytic Thrombin-Evoked VEGF Release is Dependent on P44/42 MAPKs and PAR1. Biochem Biophys Res Commun (2019) 509(2):585–9. doi: 10.1016/j.bbrc.2018.12.168 30606478

[85] Shi W Wang Z Pu J Wang R Guo Z Liu C. Changes of Blood–Brain Barrier Permeability Following Intracerebral Hemorrhage and the Therapeutic Effect of Minocycline in Rats. Acta Neurochir Suppl (2011) 110(Pt 2):61–7. doi: 10.1007/978-3-7091-0356-2_12 21125447

[86] Hashimoto T Emala CW Joshi S Mesa-Tejada R Quick CM Feng L. Abnormal Pattern of Tie-2 and Vascular Endothelial Growth Factor Receptor Expression in Human Cerebral Arteriovenous Malformations. Neurosurgery (2000) 47(4):910–9. doi: 10.1097/00006123-200010000-00022 11014431

[87] Zhang ZG Zhang L Jiang Q Zhang R Davies K Powers C. VEGF Enhances Angiogenesis and Promotes Blood-Brain Barrier Leakage in the Ischemic Brain. J Clin Invest (2000) 106(7):829–38. doi: 10.1172/JCI9369 PMC517814 11018070

[88] Hayashi T Noshita N Sugawara T Chan PH. Temporal Profile of Angiogenesis and Expression of Related Genes in the Brain After Ischemia. J Cereb Blood Flow Metab (2003) 23(2):166–80. doi: 10.1097/01.WCB.0000041283.53351.CB 12571448

[89] Jung S Moon K-S Jung T-Y Kim I-Y Lee Y-H Rhu H-H. Possible Pathophysiological Role of Vascular Endothelial Growth Factor (VEGF) and Matrix Metalloproteinases (MMPs) in Metastatic Brain Tumor-Associated Intracerebral Hemorrhage. J Neuro-Oncol (2006) 76(3):257–63. doi: 10.1007/s11060-005-6876-z 16158215

[90] Zhu W Chen W Zou D Wang L Bao C Zhan L. Thalidomide Reduces Hemorrhage of Brain Arteriovenous Malformations in a Mouse Model. Stroke (2018) 49(5):1232–40. doi: 10.1161/STROKEAHA.117.020356 PMC5916043 29593101

[91] Lee CZ Xue Z Zhu Y Yang G-Y Young WL. Matrix Metalloproteinase-9 Inhibition Attenuates Vascular Endothelial Growth Factor-Induced Intracerebral Hemorrhage. Stroke (2007) 38(9):2563–8. doi: 10.1161/STROKEAHA.106.481515 17673717

[92] Wu J Yang S Hua Y Liu W Keep R Xi G. Minocycline Attenuates Brain Edema, Brain Atrophy and Neurological Deficits After Intracerebral Hemorrhage. Acta Neurochir Suppl (2010) 106:147–50. doi: 10.1007/978-3-211-98811-4_26 19812938

[93] Wang G Li Z Li S Ren J Suresh V Xu D. Minocycline Preserves the Integrity and Permeability of BBB by Altering the Activity of DKK1–Wnt Signaling in ICH Model. Neuroscience (2019) 415:135–46. doi: 10.1016/j.neuroscience.2019.06.038 31344398

[94] Kaufmann T Strasser A Jost PJ. Fas Death Receptor Signalling: Roles of Bid and XIAP. Cell Death Differ (2012) 19(1):42. doi: 10.1038/cdd.2011.121 21959933 PMC3252833

[95] Lavrik I Krammer PH. Regulation of CD95/Fas Signaling at the DISC. Cell Death Differ (2012) 19(1):36–41. doi: 10.1038/cdd.2011.155 22075988 PMC3252827

[96] Van Herreweghe F Festjens N Declercq W Vandenabeele P. Tumor Necrosis Factor-Mediated Cell Death: To Break or to Burst, That’s the Question. Cell Mol Life Sci (2010) 67(10):1567–79. doi: 10.1007/s00018-010-0283-0 PMC11115929 20198502

[97] Sartorius U Schmitz I Krammer PH. Molecular Mechanisms of Death-Receptor-Mediated Apoptosis. Chembiochem (2001) 2(1):20–9. doi: 10.1002/1439-7633(20010105)2:1<20::AID-CBIC20>3.0.CO;2-X 11828422

[98] Brenner D Mak TW. Mitochondrial Cell Death Effectors. Curr Opin Cell Biol (2009) 21(6):871–7. doi: 10.1016/j.ceb.2009.09.004 19822411

[99] Chalah A Khosravi-Far R. The Mitochondrial Death Pathway. Adv Exp Med Biol (2008) 615:25–45. doi: 10.1007/978-1-4020-6554-5_3 18437890

[100] Lindsay J Degli Esposti M Gilmore AP. Bcl-2 Proteins and Mitochondria—Specificity in Membrane Targeting for Death. Biochim Biophys Acta (BBA)-Molecular Cell Res (2011) 1813(4):532–9. doi: 10.1016/j.bbamcr.2010.10.017 21056595

[101] Ola MS Nawaz M Ahsan H. Role of Bcl-2 Family Proteins and Caspases in the Regulation of Apoptosis. Mol Cell Biochem (2011) 351(1-2):41–58. doi: 10.1007/s11010-010-0709-x 21210296

[102] Kantari C Walczak H. Caspase-8 and Bid: Caught in the Act Between Death Receptors and Mitochondria. Biochim Biophys Acta (BBA)-Molecular Cell Res (2011) 1813(4):558–63. doi: 10.1016/j.bbamcr.2011.01.026 21295084

[103] Marchi S Patergnani S Missiroli S Morciano G Rimessi A Wieckowski MR. Mitochondrial and Endoplasmic Reticulum Calcium Homeostasis and Cell Death. Cell Calcium (2018) 69:62–72. doi: 10.1016/j.ceca.2017.05.003 28515000

[104] Nakashima K Yamashita K Uesugi S Ito H. Temporal and Spatial Profile of Apoptotic Cell Death in Transient Intracerebral Mass Lesion of the Rat. J Neurotrauma (1999) 16(2):143–51. doi: 10.1089/neu.1999.16.143 10098959

[105] Qureshi AI Ling GS Khan J Suri MFK Miskolczi L Guterman LR. Quantitative Analysis of Injured, Necrotic, and Apoptotic Cells in a New Experimental Model of Intracerebral Hemorrhage. Crit Care Med (2001) 29(1):152–7. doi: 10.1097/00003246-200101000-00030 11176176

[106] Felberg RA Grotta JC Shirzadi AL Strong R Narayana P Hill-Felberg SJ. Cell Death in Experimental Intracerebral Hemorrhage: The “Black Hole” Model of Hemorrhagic Damage. Ann Neurol: Off J Am Neurol Assoc Child Neurol Soc (2002) 51(4):517–24. doi: 10.1002/ana.10160 11921058

[107] Wang X Zhu S Drozda M Zhang W Stavrovskaya IG Cattaneo E. Minocycline Inhibits Caspase-Independent and-Dependent Mitochondrial Cell Death Pathways in Models of Huntington's Disease. Proc Natl Acad Sci (2003) 100(18):10483–7. doi: 10.1073/pnas.1832501100 PMC193587 12930891

[108] Chen M Ona VO Li M Ferrante RJ Fink KB Zhu S. Minocycline Inhibits Caspase-1 and Caspase-3 Expression and Delays Mortality in a Transgenic Mouse Model of Huntington Disease. Nat Med (2000) 6(7):797–801. doi: 10.1038/77528 10888929

[109] Zhu S Stavrovskaya IG Drozda M Kim BY Ona V Li M. Minocycline Inhibits Cytochrome C Release and Delays Progression of Amyotrophic Lateral Sclerosis in Mice. Nature (2002) 417(6884):74–8. doi: 10.1038/417074a 11986668

[110] Scarabelli TM Stephanou A Pasini E Gitti G Townsend P Lawrence K. Minocycline Inhibits Caspase Activation and Reactivation, Increases the Ratio of XIAP to Smac/DIABLO, and Reduces the Mitochondrial Leakage of Cytochrome C and Smac/DIABLO. J Am Coll Cardiol (2004) 43(5):865–74. doi: 10.1016/j.jacc.2003.09.050 14998631

[111] Wang J Wei Q Wang C-Y Hill WD Hess DC Dong Z. Minocycline Up-Regulates Bcl-2 and Protects Against Cell Death in Mitochondria. J Biol Chem (2004) 279(19):19948–54. doi: 10.1074/jbc.M313629200 15004018

[112] Stirling DP Khodarahmi K Liu J McPhail LT McBride CB Steeves JD. Minocycline Treatment Reduces Delayed Oligodendrocyte Death, Attenuates Axonal Dieback, and Improves Functional Outcome After Spinal Cord Injury. J Neurosci (2004) 24(9):2182–90. doi: 10.1523/JNEUROSCI.5275-03.2004 PMC6730425 14999069

[113] Maier K Merkler D Gerber J Taheri N Kuhnert AV Williams SK. Multiple Neuroprotective Mechanisms of Minocycline in Autoimmune CNS Inflammation. Neurobiol Dis (2007) 25(3):514–25. doi: 10.1016/j.nbd.2006.10.022 17239606

[114] Wu Z Zou X Zhu W Mao Y Chen L Zhao F. Minocycline is Effective in Intracerebral Hemorrhage by Inhibition of Apoptosis and Autophagy. J Neurol Sci (2016) 371:88–95. doi: 10.1016/j.jns.2016.10.025 27871457

[115] Li Z Liu Y Wei R Khan S Xue M Yong VW. The Combination of Deferoxamine and Minocycline Strengthens Neuroprotective Effect on Acute Intracerebral Hemorrhage in Rats. Neurol Res (2021) 43(10):854–64. doi: 10.1080/01616412.2021.1939487 34107863

[116] Amenta J Brocher S. Minireview: Mechanisms of Protein Turnover in Cultured Cells. Life Sci (1981) 28(11):1195–208. doi: 10.1016/0024-3205(81)90444-6 7015046

[117] Glaumann H Ericsson JL Marzella L. Mechanisms of Intralysosomal Degradation With Special Reference to Autophagocytosis and Heterophagocytosis of Cell Organelles. Int Rev Cytol (1981) 73:149–82. doi: 10.1016/S0074-7696(08)61288-7 7028659

[118] Bobinger T Burkardt P B Huttner H Manaenko A. Programmed Cell Death After Intracerebral Hemorrhage. Curr Neuropharmacol (2018) 16(9):1267–81. doi: 10.2174/1570159X15666170602112851 PMC6251052 28571544

[119] He Y Wan S Hua Y Keep RF Xi G. Autophagy After Experimental Intracerebral Hemorrhage. J Cereb Blood Flow Metab (2008) 28(5):897–905. doi: 10.1038/sj.jcbfm.9600578 17987045

[120] Kanthasamy A Anantharam V Ali SF Kanthasamy A. Methamphetamine Induces Autophagy and Apoptosis in a Mesencephalic Dopaminergic Neuronal Culture Model: Role of Cathepsin-D in Methamphetamine-Induced Apoptotic Cell Death. Ann New York Acad Sci (2006) 1074(1):234–44. doi: 10.1196/annals.1369.022 17105920

[121] Todde V Veenhuis M van der Klei IJ. Autophagy: Principles and Significance in Health and Disease. Biochim Biophys Acta (BBA)-Molecular Basis Dis (2009) 1792(1):3–13. doi: 10.1016/j.bbadis.2008.10.016 19022377

[122] Kabeya Y Mizushima N Ueno T Yamamoto A Kirisako T Noda T. LC3, a Mammalian Homologue of Yeast Apg8p, is Localized in Autophagosome Membranes After Processing. EMBO J (2000) 19(21):5720–8. doi: 10.1093/emboj/19.21.5720 PMC305793 11060023

[123] Mizushima N. Methods for Monitoring Autophagy. Int J Biochem Cell Biol (2004) 36(12):2491–502. doi: 10.1016/j.biocel.2004.02.005 15325587

[124] Sun J Shigemi H Cao M Qin E Tang J Shen J. Minocycline Induces Autophagy and Inhibits Cell Proliferation in LPS-Stimulated THP-1 Cells. BioMed Res Int (2020) 2020:5459209. doi: 10.1155/2020/5459209 32766308 PMC7387962

[125] Liu W-T Lin C-H Hsiao M Gean P-W. Minocycline Inhibits the Growth of Glioma by Inducing Autophagy. Autophagy (2011) 7(2):166–75. doi: 10.4161/auto.7.2.14043 21079420

[126] Zhang E Zhao X Zhang L Li N Yan J Tu K. Minocycline Promotes Cardiomyocyte Mitochondrial Autophagy and Cardiomyocyte Autophagy to Prevent Sepsis-Induced Cardiac Dysfunction by Akt/mTOR Signaling. Apoptosis (2019) 24(3-4):369–81. doi: 10.1007/s10495-019-01521-3 30756206

[127] Xiao S-G Dong W-B Cheng M Ye X-D Zheng G-L. Autophagy Activation Contributes to the Protection of Minocycline Against Oxygen-Glucose Deprivation and Reperfusion in PC12 Cells. Chin J Clin Pharmacol Ther (2015) 20(2):145.

[128] Kopitar-Jerala N. Innate Immune Response in Brain, NF-Kappa B Signaling and Cystatins. Front Mol Neurosci (2015) 8:73. doi: 10.3389/fnmol.2015.00073 26696821 PMC4673337

[129] Kate S Jurg T. The Inflammasomes. Cell (2010) 140:821–32. doi: 10.1016/j.cell.2010.01.040 20303873

[130] Johann S Heitzer M Kanagaratnam M Goswami A Rizo T Weis J. NLRP3 Inflammasome is Expressed by Astrocytes in the SOD1 Mouse Model of ALS and in Human Sporadic ALS Patients. Glia (2015) 63(12):2260–73. doi: 10.1002/glia.22891 26200799

[131] Tong Y Ding Z-H Zhan F-X Cai L Yin X Ling J-L. The NLRP3 Inflammasome and Stroke. Int J Clin Exp Med (2015) 8(4):4787–94. PMC4483817 26131053

[132] Zhao X Gu C Yan C Zhang X Li Y Wang L. NALP3-Inflammasome-Related Gene Polymorphisms in Patients With Prehypertension and Coronary Atherosclerosis. BioMed Res Int (2016) 2016:7395627. doi: 10.1155/2016/7395627 27446957 PMC4944040

[133] Minutoli L Puzzolo D Rinaldi M Irrera N Marini H Arcoraci V. ROS-Mediated NLRP3 Inflammasome Activation in Brain, Heart, Kidney, and Testis Ischemia/Reperfusion Injury. Oxid Med Cell Longevity (2016) 2016:2183026. doi: 10.1155/2016/2183026 PMC4835650 27127546

[134] Fann DY-W Santro T Manzanero S Widiapradja A Cheng Y-L Lee S-Y. Intermittent Fasting Attenuates Inflammasome Activity in Ischemic Stroke. Exp Neurol (2014) 257:114–9. doi: 10.1016/j.expneurol.2014.04.017 24805069

[135] Liang Y Jing X Zeng Z Bi W Chen Y Wu X. Rifampicin Attenuates Rotenone-Induced Inflammation *via* Suppressing NLRP3 Inflammasome Activation in Microglia. Brain Res (2015) 1622:43–50. doi: 10.1016/j.brainres.2015.06.008 26086368

[136] Pan Y Chen X-Y Zhang Q-Y Kong L-D. Microglial NLRP3 Inflammasome Activation Mediates IL-1β-Related Inflammation in Prefrontal Cortex of Depressive Rats. Brain Behav Immun (2014) 41:90–100. doi: 10.1016/j.bbi.2014.04.007 24859041

[137] Chen X He W-T Hu L Li J Fang Y Wang X. Pyroptosis is Driven by non-Selective Gasdermin-D Pore and its Morphology is Different From MLKL Channel-Mediated Necroptosis. Cell Res (2016) 26(9):1007–20. doi: 10.1038/cr.2016.100 PMC5034106 27573174

[138] Wallach D Kang T-B Dillon CP Green DR. Programmed Necrosis in Inflammation: Toward Identification of the Effector Molecules. Science (2016) 352(6281):aaf2154. doi: 10.1126/science.aaf2154 27034377

[139] Wu B Ma Q Khatibi N Chen W Sozen T Cheng O. Ac-YVAD-CMK Decreases Blood–Brain Barrier Degradation by Inhibiting Caspase-1 Activation of Interleukin-1β in Intracerebral Hemorrhage Mouse Model. Trans Stroke Res (2010) 1(1):57–64. doi: 10.1007/s12975-009-0002-z PMC2892994 20596246

[140] Feng L Chen Y Ding R Fu Z Yang S Deng X. P2X7R Blockade Prevents NLRP3 Inflammasome Activation and Brain Injury in a Rat Model of Intracerebral Hemorrhage: Involvement of Peroxynitrite. J Neuroinflamm (2015) 12(1):1–17. doi: 10.1186/s12974-015-0409-2 PMC4609067 26475134

[141] Ma Q Chen S Hu Q Feng H Zhang JH Tang J. NLRP3 Inflammasome Contributes to Inflammation After Intracerebral Hemorrhage. Ann Neurol (2014) 75(2):209–19. doi: 10.1002/ana.24070 PMC4386653 24273204

[142] Ahsan H. 3-Nitrotyrosine: A Biomarker of Nitrogen Free Radical Species Modified Proteins in Systemic Autoimmunogenic Conditions. Hum Immunol (2013) 74(10):1392–9. doi: 10.1016/j.humimm.2013.06.009 23777924

[143] Wanyong Y Zefeng T Xiufeng X Dawei D Xiaoyan L Ying Z. Tempol Alleviates Intracerebral Hemorrhage-Induced Brain Injury Possibly by Attenuating Nitrative Stress. Neuroreport (2015) 26(14):842–9. doi: 10.1097/WNR.0000000000000434 26237245

[144] Yang Z Zhong L Xian R Yuan B. MicroRNA-223 Regulates Inflammation and Brain Injury *via* Feedback to NLRP3 Inflammasome After Intracerebral Hemorrhage. Mol Immunol (2015) 65(2):267–76. doi: 10.1016/j.molimm.2014.12.018 25710917

[145] Babu R Bagley JH Di C Friedman AH Adamson C. Thrombin and Hemin as Central Factors in the Mechanisms of Intracerebral Hemorrhage–Induced Secondary Brain Injury and as Potential Targets for Intervention. Neurosurgical Focus (2012) 32(4):E8. doi: 10.3171/2012.1.FOCUS11366 22463118

[146] Mohan S Glushakov AV deCurnou A Narumiya S Doré S. Contribution of PGE2 EP1 Receptor in Hemin-Induced Neurotoxicity. Front Mol Neurosci (2013) 6:31. doi: 10.3389/fnmol.2013.00031 24109429 PMC3791386

[147] Yang F Zhu W Cai X Zhang W Yu Z Li X. Minocycline Alleviates NLRP3 Inflammasome-Dependent Pyroptosis in Monosodium Glutamate-Induced Depressive Rats. Biochem Biophys Res Commun (2020) 526(3):553–9. doi: 10.1016/j.bbrc.2020.02.149 32245616

[148] Poon CC Sarkar S Yong VW Kelly JJ. Glioblastoma-Associated Microglia and Macrophages: Targets for Therapies to Improve Prognosis. Brain (2017) 140(6):1548–60. doi: 10.1093/brain/aww355 28334886

[149] Bai Q Xue M Yong VW. Microglia and Macrophage Phenotypes in Intracerebral Haemorrhage Injury: Therapeutic Opportunities. Brain (2020) 143(5):1297–314. doi: 10.1093/brain/awz393 31919518

[150] Yang J Ding S Huang W Hu J Huang S Zhang Y. Interleukin-4 Ameliorates the Functional Recovery of Intracerebral Hemorrhage Through the Alternative Activation of Microglia/Macrophage. Front Neurosci (2016) 10:61. doi: 10.3389/fnins.2016.00061 27013935 PMC4781843

[151] Lin L Yihao T Zhou F Yin N Qiang T Haowen Z. Inflammatory Regulation by Driving Microglial M2 Polarization: Neuroprotective Effects of Cannabinoid Receptor-2 Activation in Intracerebral Hemorrhage. Front Immunol (2017) 8:112. doi: 10.3389/fimmu.2017.00112 28261199 PMC5306140

[152] Zhou K Zhong Q Wang Y-C Xiong X-Y Meng Z-Y Zhao T. Regulatory T Cells Ameliorate Intracerebral Hemorrhage-Induced Inflammatory Injury by Modulating Microglia/Macrophage Polarization Through the IL-10/Gsk3β/PTEN Axis. J Cereb Blood Flow Metab (2017) 37(3):967–79. doi: 10.1177/0271678X16648712 PMC5363473 27174997

[153] Xue M Del Bigio MR. Immune Pre-Activation Exacerbates Hemorrhagic Brain Injury in Immature Mouse Brain. J Neuroimmunol (2005) 165(1-2):75–82. doi: 10.1016/j.jneuroim.2005.04.016 15964638

[154] Chen S Zhao L Sherchan P Ding Y Yu J Nowrangi D. Activation of Melanocortin Receptor 4 With RO27-3225 Attenuates Neuroinflammation Through AMPK/JNK/p38 MAPK Pathway After Intracerebral Hemorrhage in Mice. J Neuroinflamm (2018) 15(1):106. doi: 10.1186/s12974-018-1140-6 PMC5896146 29642894

[155] Zhang Z Liu Y Huang Q Su Y Zhang Y Wang G. NF-κb Activation and Cell Death After Intracerebral Hemorrhage in Patients. Neurol Sci (2014) 35(7):1097–102. doi: 10.1007/s10072-014-1657-0 24510152

[156] Tikka T Fiebich BL Goldsteins G Keinänen R Koistinaho J. Minocycline, a Tetracycline Derivative, is Neuroprotective Against Excitotoxicity by Inhibiting Activation and Proliferation of Microglia. J Neurosci (2001) 21(8):2580–8. doi: 10.1523/JNEUROSCI.21-08-02580.2001 PMC6762519 11306611

[157] Kim S-S Kong P-J Kim B-S Sheen D-H Nam S-Y Chun W. Inhibitory Action of Minocycline on Lipopolysaccharide-Lnduced Release of Nitric Oxide and Prostaglandin E2 in BV2 Microglial Cells. Arch Pharmacal Res (2004) 27(3):314. doi: 10.1007/BF02980066 15089037

[158] Kobayashi K Imagama S Ohgomori T Hirano K Uchimura K Sakamoto K. Minocycline Selectively Inhibits M1 Polarization of Microglia. Cell Death Dis (2013) 4(3):e525–e. doi: 10.1038/cddis.2013.54 PMC3613832 23470532

[159] Wu J Yang S Xi G Fu G Keep RF Hua Y. Minocycline Reduces Intracerebral Hemorrhage-Induced Brain Injury. Neurol Res (2009) 31(2):183–8. doi: 10.1179/174313209X385680 PMC2659336 19061541

[160] Yang H Gao XJ Li YJ Su JB TZ E Zhang X. Minocycline Reduces Intracerebral Hemorrhage–Induced White Matter Injury in Piglets. CNS Neurosci Ther (2019) 25(10):1195–206. doi: 10.1111/cns.13220 PMC6776747 31556245

[161] Bao X Wu G Hu S Huang F. Poly (ADP-Ribose) Polymerase Activation and Brain Edema Formation by Hemoglobin After Intracerebral Hemorrhage in Rats. Acta Neurochir Suppl (2008) 105:23–7. doi: 10.1007/978-3-211-09469-3_5 19066076

[162] Imai T Tsuji S Matsubara H Ohba T Sugiyama T Nakamura S. Deferasirox, a Trivalent Iron Chelator, Ameliorates Neuronal Damage in Hemorrhagic Stroke Models. Naunyn-Schmiedeberg's Arch Pharmacol (2021) 394(1):73–84. doi: 10.1007/s00210-020-01963-6 32808069

[163] Tao R Kim SH Honbo N Karliner JS Alano CC. Minocycline Protects Cardiac Myocytes Against Simulated Ischemia-Reperfusion Injury by Inhibiting Poly (ADP-Ribose) Polymerase-1. J Cardiovasc Pharmacol (2010) 56(6):659. doi: 10.1097/FJC.0b013e3181faeaf0 20881608 PMC3064957

[164] Alano CC Kauppinen TM Valls AV Swanson RA. Minocycline Inhibits Poly (ADP-Ribose) Polymerase-1 at Nanomolar Concentrations. Proc Natl Acad Sci (2006) 103(25):9685–90. doi: 10.1073/pnas.0600554103 PMC1480467 16769901

[165] Nicolescu AC Holt A Kandasamy AD Pacher P Schulz R. Inhibition of Matrix Metalloproteinase-2 by PARP Inhibitors. Biochem Biophys Res Commun (2009) 387(4):646–50. doi: 10.1016/j.bbrc.2009.07.080 PMC2756481 19619515

[166] Zhao H Zhang J Hong G. Minocycline Improves Cardiac Function After Myocardial Infarction in Rats by Inhibiting Activation of PARP-1. Biomed Pharmacother (2018) 97:1119–24. doi: 10.1016/j.biopha.2017.10.053 29136949

[167] Shultz RB Zhong Y. Minocycline Targets Multiple Secondary Injury Mechanisms in Traumatic Spinal Cord Injury. Neural Regener Res (2017) 12(5):702. doi: 10.4103/1673-5374.206633 PMC5461601 28616020

[168] Parenti A Indorato B Paccosi S. Minocycline Affects Human Neutrophil Respiratory Burst and Transendothelial Migration. Inflammation Res (2017) 66(2):107–9. doi: 10.1007/s00011-016-0999-x 27757474

[169] Kloppenburg M Verweij C Miltenburg A Verhoeven A Daha M Dijkmans B. The Influence of Tetracyclines on T Cell Activation. Clin Exp Immunol (1995) 102(3):635–41. doi: 10.1111/j.1365-2249.1995.tb03864.x PMC1553388 8536384

[170] Zou X Wu Z Zhu W Chen L Mao Y Zhao F. Effectiveness of Minocycline in Acute White Matter Injury After Intracerebral Hemorrhage. J Neurosurg (2016) 126(6):1855–62. doi: 10.3171/2016.5.JNS152670 27494818

[171] Lin S Zhang Y Dodel R Farlow MR Paul SM Du Y. Minocycline Blocks Nitric Oxide-Induced Neurotoxicity by Inhibition P38 MAP Kinase in Rat Cerebellar Granule Neurons. Neurosci Lett (2001) 315(1-2):61–4. doi: 10.1016/S0304-3940(01)02324-2 11711215

[172] Rosenzweig R Nillegoda NB Mayer MP Bukau B. The Hsp70 Chaperone Network. Nat Rev Mol Cell Biol (2019) 20(11):665–80. doi: 10.1038/s41580-019-0133-3 31253954

[173] Freeman RS Burch RL Crowder RJ Lomb DJ Schoell MC Straub JA. NGF Deprivation-Induced Gene Expression: After Ten Years, Where do We Stand? Prog Brain Res (2004) 146:111–26. doi: 10.1016/S0079-6123(03)46008-1 14699960

[174] Pu J Shi W Wang Z Wang R Guo Z Liu C. Effects of Minocycline on the Expression of NGF and HSP70 and its Neuroprotection Role Following Intracerebral Hemorrhage in Rats. J Biomed Res (2011) 25(4):292–8. doi: 10.1016/S1674-8301(11)60040-7 PMC3597072 23554704

[175] Zhang R Xue M Yong VW. Central Nervous System Tissue Regeneration After Intracerebral Hemorrhage: The Next Frontier. Cells (2021) 10(10):2513. doi: 10.3390/cells10102513 34685493 PMC8534252

[176] Miao H Li R Han C Lu X Zhang H. Minocycline Promotes Posthemorrhagic Neurogenesis *via* M2 Microglia Polarization *via* Upregulation of the TrkB/BDNF Pathway in Rats. J Neurophysiol (2018) 120(3):1307–17. doi: 10.1152/jn.00234.2018 29790836

[177] Yong HY Rawji KS Ghorbani S Xue M Yong VW. The Benefits of Neuroinflammation for the Repair of the Injured Central Nervous System. Cell Mol Immunol (2019) 16(6):540–6. doi: 10.1038/s41423-019-0223-3 PMC6804643 30874626

[178] Lei C Wu B Cao T Liu M Hao Z. Brain Recovery Mediated by Toll-Like Receptor 4 in Rats After Intracerebral Hemorrhage. Brain Res (2016) 1632:1–8. doi: 10.1016/j.brainres.2015.11.045 26657742

[179] Jing C Bian L Wang M Keep RF Xi G Hua Y. Enhancement of Hematoma Clearance With CD47 Blocking Antibody in Experimental Intracerebral Hemorrhage. Stroke (2019) 50(6):1539–47. doi: 10.1161/STROKEAHA.118.024578 PMC6538472 31084334

[180] Bassett B Subramaniyam S Fan Y Varney S Pan H Carneiro AMD. Minocycline Alleviates Depression-Like Symptoms by Rescuing Decrease in Neurogenesis in Dorsal Hippocampus *via* Blocking Microglia Activation/Phagocytosis. Brain Behav Immun (2021) 91:519–30. doi: 10.1016/j.bbi.2020.11.009 33176182

[181] Hashemi-Monfared A Firouzi M Bahrami Z Zahednasab H Harirchian MH. Minocycline Decreases CD36 and Increases CD44 in LPS-Induced Microglia. J Neuroimmunol (2018) 317:95–9. doi: 10.1016/j.jneuroim.2018.01.010 29395319

[182] Szymanska A Biernaskie J Laidley D Granter-Button S Corbett D. Minocycline and Intracerebral Hemorrhage: Influence of Injury Severity and Delay to Treatment. Exp Neurol (2006) 197(1):189–96. doi: 10.1016/j.expneurol.2005.09.011 16259983

[183] Wasserman JK Schlichter LC. Neuron Death and Inflammation in a Rat Model of Intracerebral Hemorrhage: Effects of Delayed Minocycline Treatment. Brain Res (2007) 1136:208–18. doi: 10.1016/j.brainres.2006.12.035 17223087

[184] Wasserman JK Zhu X Schlichter LC. Evolution of the Inflammatory Response in the Brain Following Intracerebral Hemorrhage and Effects of Delayed Minocycline Treatment. Brain Res (2007) 1180:140–54. doi: 10.1016/j.brainres.2007.08.058 17919462

[185] Kohler E Prentice DA Bates TR Hankey GJ Claxton A van Heerden J. Intravenous Minocycline in Acute Stroke: A Randomized, Controlled Pilot Study and Meta-Analysis. Stroke (2013) 44(9):2493–9. doi: 10.1161/STROKEAHA.113.000780 23868273

[186] Xu L Fagan SC Waller JL Edwards D Borlongan CV Zheng J. Low Dose Intravenous Minocycline is Neuroprotective After Middle Cerebral Artery Occlusion-Reperfusion in Rats. BMC Neurol (2004) 4(1):1–7. doi: 10.1186/1471-2377-4-7 15109399 PMC415551

[187] Fouda AY Newsome AS Spellicy S Waller JL Zhi W Hess DC. Minocycline in Acute Cerebral Hemorrhage: An Early Phase Randomized Trial. Stroke (2017) 48(10):2885–7. doi: 10.1161/STROKEAHA.117.018658 28887388

[188] Strickland BA Bakhsheshian J Emmanuel B Amar A Giannotta SL Russin JJ. Neuroprotective Effect of Minocycline Against Acute Brain Injury in Clinical Practice: A Systematic Review. J Clin Neurosci (2021) 86:50–7. doi: 10.1016/j.jocn.2021.01.005 33775346

[189] Chang J Kim-Tenser M Emanuel B Jones G Chapple K Alikhani A. Minocycline and Matrix Metalloproteinase Inhibition in Acute Intracerebral Hemorrhage: A Pilot Study. Eur J Neurol (2017) 24(11):1384–91. doi: 10.1111/ene.13403 28929560

[190] Malhotra K Chang JJ Khunger A Blacker D Switzer JA Goyal N. Minocycline for Acute Stroke Treatment: A Systematic Review and Meta-Analysis of Randomized Clinical Trials. J Neurol (2018) 265(8):1871–9. doi: 10.1007/s00415-018-8935-3 29948247

[191] Xue M Yong VW. Neuroinflammation in Intracerebral Haemorrhage: Immunotherapies With Potential for Translation. Lancet Neurol (2020) 19(12):1023–32. doi: 10.1016/S1474-4422(20)30364-1 33212054

[192] Luo T Guo T Yang Q Hao S Wang J Cheng Z. *In Situ* Hydrogels Enhancing Postoperative Functional Recovery by Reducing Iron Overload After Intracerebral Haemorrhage. Int J Pharmaceutics (2017) 534(1-2):179–89. doi: 10.1016/j.ijpharm.2017.10.010 28987454

